# Characterization of Clinical Presentation, Etiology, and Antibiotic Sensitivity Patterns in Neonatal Septicemia: A Comprehensive Analysis of Bacterial Isolates

**DOI:** 10.7759/cureus.63259

**Published:** 2024-06-26

**Authors:** Naveen Sharma, Surinder Singh, Bhagat Ram Thakur, Sandesh Guleria, Pratyaksha Pandit

**Affiliations:** 1 Department of Pediatrics, Indira Gandhi Medical College and Hospital, Shimla, IND; 2 Department of Community Medicine, Uttar Pradesh University of Medical Sciences, Saifai, IND

**Keywords:** newborn, septicemia, risk factors, lons, eons, antibiotic resistance (abr), neonatal septicemia

## Abstract

Background: Neonatal septicemia remains a significant healthcare challenge, particularly in resource-limited settings, with both early-onset neonatal septicemia (EONS) and late-onset neonatal septicemia (LONS) presentations contributing to morbidity and mortality. This study aimed to characterize the clinico-etiological profile and antibiotic susceptibility patterns of neonatal septicemia in a tertiary care setting in north India.

Methodology: An analytical cross-sectional study was conducted from March 2021 to February 2022, encompassing neonates admitted to the Department of Pediatrics with suspected neonatal septicemia, confirmed by positive blood cultures.

Results: A total of 96 neonates were included, predominantly male (71.9%). Gram-negative bacteria constituted 61.6% of isolates, and the most common organism isolated was non-lactose fermenter group (38.4%) followed by coagulase-negative staphylococci (CoNS) (33.4%). Non-lactose fermenter group bacteria were prominent in EONS cases (44.6%), while CoNS predominated in LONS cases (51.6%). Birthplace, birth weight, and perinatal score were significantly associated with both EONS and LONS. Linezolid exhibited high efficacy against gram-positive bacteria, while ciprofloxacin and meropenem demonstrated effectiveness against various gram-negative pathogens. Methicillin-resistant *Staphylococcus aureus* (MRSA) strains exhibited resistance to all the antibiotics used in the study except for linezolid.

Conclusion: These findings underscore the importance of tailored empirical therapy guided by local epidemiological data to optimize clinical outcomes and mitigate antimicrobial resistance.

## Introduction

Neonatal sepsis refers to an infection involving bloodstream among neonates and is further categorized into early and late-onset sepsis [1]. It remains a significant cause of morbidity and mortality in the neonatal population, particularly in resource-limited settings. Despite advances in perinatal care and antimicrobial therapy, neonatal sepsis continues to challenge healthcare providers globally. The distinction between early-onset neonatal septicemia (EONS), typically occurring within the first 72 hours of life, and late-onset neonatal septicemia (LONS), manifesting after 72 hours, has important clinical implications due to variations in etiology and management strategies [2]. EONS is generally caused by pathogens from the maternal genitourinary system while LONS usually occurs due to the transmission of pathogens from the surrounding environment. 

The pathogenesis of neonatal septicemia involves a complex interplay between host factors, microbial virulence, and environmental exposures [3]. Bacterial pathogens, primarily originating from the maternal genital tract, colonize the neonatal skin and mucous membranes during birth, with subsequent invasion leading to septicemia [4]. Common causative organisms include gram-positive bacteria such as *Staphylococcus aureus* and *Streptococcus agalactiae* (Group B *Streptococcus*, GBS), as well as gram-negative bacteria including *Escherichia coli* and *Klebsiella* species [5].

The rising incidence of multidrug-resistant (MDR) pathogens poses a significant challenge in the management of neonatal septicemia. Empirical antimicrobial therapy must be tailored to local epidemiological data and antimicrobial susceptibility patterns to optimize clinical outcomes while minimizing the emergence of antimicrobial resistance [6]. Understanding the clinico-etiological profile and antibiotic sensitivity patterns of bacterial isolates is therefore crucial for guiding empirical therapy and informing antimicrobial stewardship initiatives.

This study aims to characterize the clinico-etiological profile of neonatal septicemia in our setting, delineating the spectrum of causative organisms and their antimicrobial susceptibility patterns. By elucidating the epidemiological trends and antimicrobial resistance profiles, this study endeavours to contribute to evidence-based guiding, rational antimicrobial use, and improving outcomes in neonatal septicemia.

## Materials and methods

An institution-based analytical cross-sectional study was conducted among neonates admitted to the Department of Pediatrics at Indira Gandhi Medical College (IGMC) and Kamla Nehru Hospital (KNH) with clinical features suggestive of neonatal septicemia and subsequently documented by positive blood culture. The study was undertaken from March 2021 to February 2022. Neonates with gross congenital anomalies and whose mothers refused to give consent were excluded from the study. The confidentiality of each study participant was maintained throughout the study. The study was approved by the Institutional Ethical Committee, IGMC (approval number: HFW(MC-2)B(12)ETHICS/2021/14453), and informed consent was taken from the study participants.

Based on signs, symptoms, and history, blood culture was done of suspected cases and neonates with positive cultures during the mentioned period were consecutively included in the study. Cases presenting within 72 hours of life were categorized as EONS and the neonates presenting after 72 hours of life were labelled as cases of LONS. Taking the prevalence of blood culture positive for isolates as 26%, 95% confidence level and 9% absolute error, the final sample size calculated was 96 [7].

Blood culture was performed by the BACTEC™ system (Becton, Dickinson and Company, Franklin Lakes, New Jersey, United States) [8] and antibiotic sensitivity was done by the method of Bauer and Kirby (1966) [9] for manual sensitivity or BD Phoenix™ automated identification and susceptibility system (Becton, Dickinson and Company). The weight of the baby was measured at the time of admission. Antibiotics used in the study were linezolid, gentamycin, cefotaxime, ciprofloxacin, ampicillin, piperacillin-tazobactam, meropenem, ceftazidime, vancomycin, amikacin. The perinatal score was calculated and categorized into low, moderate, and high. A pre-designed, pre-tested semi-structured questionnaire was used to gather data.

Data was entered in a Microsoft Excel sheet (Microsoft Corporation, Redmond, Washington, United States) and analyzed using IBM SPSS Statistics for Windows, Version 26.0 (Released 2019; IBM Corp., Armonk, New York, United States). A descriptive summary (age, gender, EONS, LONS) was presented using frequencies, percentages, graphs, mean, and standard deviation (SD). Probability (p) was calculated to test statistical significance at the 5% level of significance. Categorical variables were analyzed using the chi-square test; in case the expected frequency was found to be less than 5 in any particular cell, Fisher's exact test was used. A p-value <0.05 was considered to be statistically significant.

## Results

This study recruited 96 neonates with a median age of two days (interquartile range (IQR), 1-7), of whom 71.9% (n=69) were male and 28.1% (n=27) were female. Table 1 describes the EONS and LONS cases in the study population; 67.7% of cases were EONS and the remaining 32.3% were LONS. The highest incidence of both EONS and LONS was found in male neonates (69.2% and 77.4%, respectively).

**Table 1:** Distribution of EONS and LONS cases among study population (N=96) EONS: early-onset neonatal septicemia; LONS: late-onset neonatal septicemia

Variables	Male, n (%)	Female, n (%)	Total, n (%)
Early-onset neonatal septicemia (<72 hours)	45 (69.2)	20 (44.4)	65 (67.7)
Late-onset neonatal septicemia (>72 hours)	24 (77.4)	7 (22.5)	31 (32.3)

Table 2 describes the distribution of clinical presentation of neonates in EONS and LONS. The majority of the neonates in both groups had respiratory distress (EONS: 73.8%, LONS: 58.1%). Among neonates who had EONS, 21.5% were lethargic, 12.3% had a history of neonatal seizures, 15.4% had neonatal jaundice, and 21.5% were hypoglycemic. In neonates with LONS, 38.7% were lethargic, 16.1% had neonatal seizures, 19.4% had neonatal jaundice, and 9.7% were hypoglycemic. 

**Table 2:** Clinical presentation of study subjects (N=96) Chi-square statistics, p-value<0.05 was considered statistically significant EONS: early-onset neonatal septicemia; LONS: late-onset neonatal septicemia

Clinical presentation		EONS (n= 65)	LONS (n=31)	p-value	Phi-crammer’s V value
Respiratory distress	Yes	48 (73.8)	18 (58.1)	0.11	0.15
	No	17 (26.2)	13 (41.9)		
Lethargy	Yes	14 (21.5)	12 (38.7)	0.07	0.18
	No	51 (78.5)	19 (61.3)		
Neonatal seizures	Yes	8 (12.3)	5 (16.1)	0.60	0.05
	No	57 (87.7)	26 (83.9)		
Neonatal jaundice	Yes	10 (15.4)	6 (19.4)	0.62	0.05
	No	55 (84.6)	25 (80.6)		
Hypoglycemia	Yes	14 (21.5)	3 (9.7)	0.15	0.14
	No	51 (78.5)	28 (90.3)		

**Figure 1:**
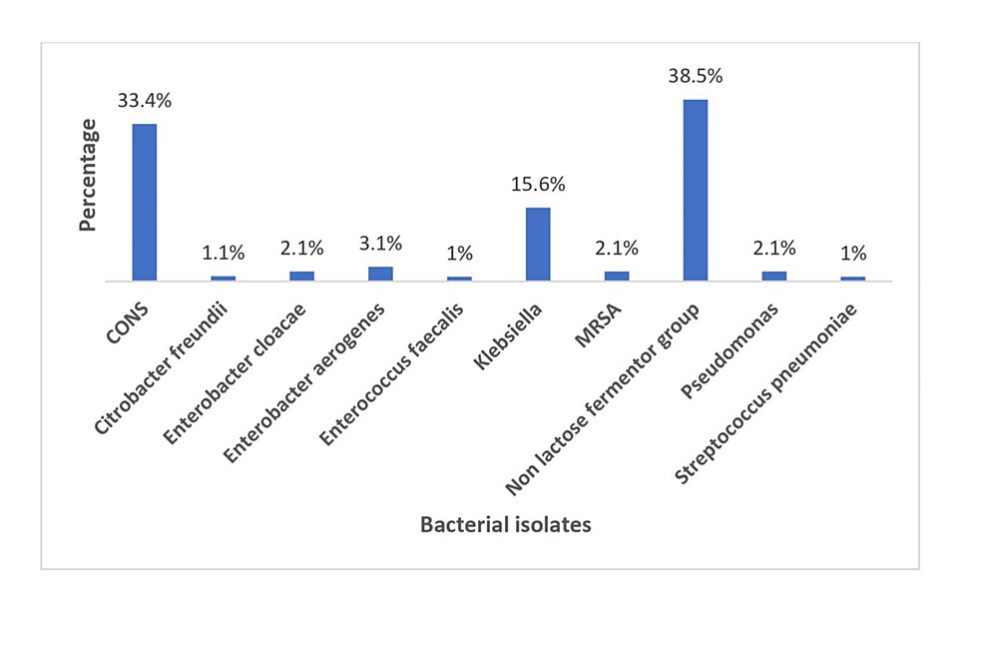
Bacteriological profile of neonatal sepsis cases CoNS: coagulase-negative staphylococci; MRSA: methicillin-resistant *Staphylococcus aureus*

The bacteriological profile showed 61.6% were gram-negative and 38.4% were gram-positive. The most common micro-organism isolated was non-lactose fermenter group (38.5%) followed by coagulase-negative staphylococci (CoNS) (33.4%), *Klebsiella* (15.6%), *Enterobacter aerogenes* (3.1%), *Enterobacter cloacae* (2.1%), methicillin-resistant *S. aureus* (MRSA) (2.1%), *Pseudomonas* (2.1%), *Citrobacter freundii* (1%), *Enterococcus faecalis* (1%), and *Streptococcus pneumoniae* (1%) (Figure 1).

Non-lactose fermenter group (44.6%) was the most common organism found to be causing EONS followed by CoNS (24.6%) and *Klebsiella* (16.9%). The most common bacterial isolate found to be causing LONS was CoNS (51.6%) followed by non-lactose fermenter group (25.8%). But this difference was not statistically significant (p-value=0.214) (Table 3).

**Table 3:** Bacteria causing neonatal septicaemia (N=96) Chi-square statistics, p-value<0.05 was considered statistically significant EONS: early-onset neonatal septicemia; LONS: late-onset neonatal septicemia; CONS: coagulase-negative staphylococci; MRSA: methicillin-resistant *Staphylococcus aureus*

Causative organism	Neonatal septicaemia		p-value
	EONS (n=65)	LONS (n=31)	
CONS	16 (24.6)	16 (51.6)	0.214
Citrobacter freundii	-	1 (3.2)	
Enterobacter cloacae	1 (1.5)	1 (3.2)	
Enterobacter aerogenes	2 (3.1)	1 (3.2)	
Enterococcus faecalis	1 (1.5)	-	
Klebsiella	11 (16.9)	4 (12.9)	
MRSA	2 (3.1)	-	
Non-lactose fermenter group	29 (44.6)	8 (25.8)	
Pseudomonas	2 (3.1)	-	
Staphylococcus hemolyticus	2 (3.1)	2 (6.5)	
Streptococcus pneumoniae	1 (1.5)	-	

No significant association was observed with respect to gender, mother's age, gestation age, and mode of delivery between EONS and LONS. In relation to various neonatal risk factors, EONS and LONS were significantly associated with place of birth (p-value: <0.001), birth weight (p-value: 0.003), and perinatal score (p-value: 0.047) (Table 4).

**Table 4:** Association between neonatal risk factors and neonatal septicemia (N=96) Chi-square statistics, p-value<0.05 was considered significant; *Fisher's exact test KNH: Kamla Nehru Hospital; LBW: low birth weight; LSCS: lower segment cesarean section; EONS: early-onset neonatal septicemia; LONS: late-onset neonatal septicemia

Variable		Neo-natal septicemia		p-value
		EONS (n=65)	LONS (n=31)	
Gender	Male	45 (69.2)	24 (77.4)	0.404
	Female	20 (30.8)	7 (22.6)	
Mother age (in years)*	<25	32 (49.2)	19 (61.2)	0.533
	25-30	21 (32.3)	8 (25.8)	
	>30	12 (18.4)	4 (12.9)	
Birthplace	Not born in KNH	18 (27.7)	21 (67.7)	<0.001
	KNH	47 (72.3)	10 (32.3)	
Gestation age	Preterm	41 (63.1)	10 (32.3)	0.005
	Term	24 (36.9)	21 (67.7	
Birth weight*	Extremely LBW	2 (3.1)	-	0.003
	Very LBW	20 (30.8)	3 (9.7)	
	LBW	29 (44.6)	10 (32.3)	
	Normal	14 (21.5)	18 (58.1)	
Mode of delivery*	Vaginal	50 (76.9)	27 (87.1)	0.242
	LSCS	15 (23.1)	4 (12.9)	
Perinatal score*	Low	31 (47.7)	22 (71)	0.047
	Moderate	19 (29.2)	4 (12.9)	
	High	15 (23.1)	5 (6.1)	

Table 5 describes the susceptibility of the bacteriological isolates to different antibiotics. The most effective antibiotic against gram-positive bacteria was found to be linezolid followed by vancomycin. Ampicillin was the least effective antibiotic both among gram-positive and gram-negative isolates. Among *Acinetobacter* isolates, meropenem and ciprofloxacin were found to be 100% sensitive. Isolates of *Pseudomonas* were sensitive to only ciprofloxacin and cefotaxime. Ciprofloxacin was effective against *E. cloacae* while linezolid, vancomycin, gentamycin, and ampicillin were effective against *E. faecalis*. Only ciprofloxacin (33.3%) was effective against *E. aerogenes*. All the CoNS strains were susceptible towards linezolid. Among *Klebsiella* isolates, the most effective antibiotic was ciprofloxacin followed by meropenem. Linezolid was effective against both *Staphylococcus haemolyticus* and *S. pneumoniae*. Gentamycin, ciprofloxacin, and cefotaxime were effective against *Citrobacter* isolates. MRSA isolates were resistant to all antibiotics, except linezolid (100%).

**Table 5:** Antibiotic susceptibility pattern of isolates (N=96) - : resistant; NA: not applicable CoNS: coagulase-negative staphylococci; MRSA: methicillin-resistant *Staphylococcus aureus*

Antibiotics	*Acinetobacter* (n=10), n (%)	*Burkholderia *(n=8), n (%)	CoNS (n=28), n (%)	*Citrobacter* (n=1), n (%)	*Enterobacter cloacae* (n=2), n (%)	*Enterobacter aerogenes* (n=3), n (%)	*Enterococcus faecalis* (n=1), n (%)	*Klebsiella* (n=15), n (%)	MRSA (n=2), n (%)	Non-lactose fermenter (n=19), n (%)	*Pseudomonas* (n=2), n (%)	*Staphylococcus haemolyticus *(n=4), n (%)	*Streptococcus pneumoniae* (n=1), n (%)
Linezolid	5 (50)	3 (37.5)	28 (100)	NA	NA	NA	1 (100)	3 (20)	2 (100)	6 (31.6)	NA	4 (100)	1 (100)
Meropenem	10 (100)	6 (75)	16 (57.1)	-	1 (50)	-	-	8 (53.3)	-	13 (68.4)	-	1 (25)	-
Ceftazidime	-	7 (87.5)	2 (7.1)	-	1 (50)	-	-	-	NA	1 (5.3)	-	1 (25)	NA
Gentamycin	-	-	7 (25)	1 (100)	-	-	1 (100)	4 (26.7)	NA	3 (15.8)	-	NA	NA
Ciprofloxacin	10 (100)	3 (37.5)	18 (64.3)	1 (100)	2 (100)	1 (33.3)	-	10 (66.6)	-	16 (84.2)	2 (100)	2 (50)	-
Cefotaxime	2 (20)	-	4 (14.3)	1 (100)	-	-	-	1 (6.7)	-	5 (26.3)	2 (100)	-	-
Vancomycin	1 (10)	2 (25)	11 (29.3)	NA	NA	NA	1 (100)	1 (6.7)	-	NA	NA	2 (50)	1 (100)
Amikacin	4 (40)	-	2 (7.1)	-	1 (50)	-	-	2 (13.3)	-	-	-	-	-
Ampicillin	-	-	-	-	-	-	1 (100)	-	NA	-	-	NA	NA
Piperacillin-tazobactam	5 (50)	2 (25)	2 (7.1)	-	1 (50)	-	-	3 (20)	-	8 (42.1)	-	-	-

## Discussion

Neonatal septicemia represents a critical challenge in neonatal care, necessitating a thorough understanding of its clinico-etiological profile and antimicrobial susceptibility patterns to guide effective management strategies. In this study, we investigated the characteristics of EONS and LONS, shedding light on the prevalence of causative organisms and their antibiotic sensitivity profiles.

Our findings reveal a predominance of EONS comprising 67.7% of cases, consistent with previous reports indicating a higher incidence within the first 72 hours of life [2,10]. Comparing the results to an earlier study published in the same institute showed a fall in the incidence of neonatal septicemia [5]. This underscores the importance of early recognition and prompt initiation of appropriate antimicrobial therapy in the neonatal period. Interestingly, no significant gender predilection was observed in the distribution of EONS and LONS, highlighting the non-discriminatory nature of neonatal septicemia with regard to sex.

The bacteriological profile identified in our study reflects a notable predominance of gram-negative organisms, accounting for 61.6% of isolates. This aligns with global trends indicating an increasing prevalence of gram-negative pathogens in neonatal sepsis, posing significant therapeutic challenges due to their propensity for multidrug resistance [11,12]. A study by Rana et al. at B J Medical College, Ahmedabad, reported similar results, with *Klebsiella* species (37.25%) and CoNS (29.41%) being the most common culture isolates [13]. Rashmi et al. also documented similar results, with methicillin-resistant CoNS being the most common isolate (32.47%) in culture-positive neonates [14]. Notably, CoNS emerged as the most frequently isolated organism, consistent with its well-established role as a major pathogen in neonatal septicaemia, particularly associated with indwelling medical devices and nosocomial transmission [4].

Overall, due to increased survival and prolonged hospitalization of low birth weight (LBW) babies, who require frequent invasive procedures, and the use of higher antibiotics, the incidence of CoNS which was previously thought to be non-pathogenic has increased over the years in neonatal intensive care unit (NICU). Furthermore, our analysis delineated distinct microbial profiles associated with EONS and LONS. Non-lactose fermenter organisms, notably CoNS, were prominently implicated in both EONS and LONS, underscoring their clinical significance across the neonatal period. *Klebsiella* species exhibited a higher prevalence in LONS cases, suggesting a potential shift in microbial aetiology beyond the immediate perinatal period. These findings emphasize the importance of tailored empirical therapy based on the timing of sepsis onset and local epidemiological trends.

In terms of antimicrobial susceptibility patterns, our study identified notable variations in antibiotic efficacy against different bacterial isolates. Linezolid and vancomycin demonstrated superior activity against gram-positive pathogens, consistent with their established role as first-line agents in the management of gram-positive neonatal septicemia [11]. Patel et al. documented gentamicin (100%), vancomycin (96.87%), and ciprofloxacin (90.9%) as the most sensitive drugs for gram-positive organisms [15]. Conversely, in concordance with existing literature [16], ampicillin exhibited limited effectiveness, highlighting the need for judicious antibiotic selection to minimize the risk of treatment failure and antimicrobial resistance emergence. Among gram-negative isolates, meropenem and ciprofloxacin emerged as key therapeutic options against *Acinetobacter* spp., reflecting their broad-spectrum activity and clinical utility in critically ill neonates. Existing literature has also reported that most of the gram-negative organisms were sensitive to amikacin and ciprofloxacin [17]. However, the emergence of MDR pathogens underscores the imperative for antimicrobial stewardship initiatives to optimize antibiotic use and mitigate resistance.

Table 6 gives a comparison with the published literature.

**Table 6:** Comparison with published literature MRCoNS: methicillin-resistant coagulase-negative staphylococci; CoNS: coagulase-negative staphylococci

Serial No.	Study Name	Year	Isolates
1.	Rana et al. [13]	2016	*Klebsiella* (37.2%), CoNS (29.4%)
2.	Rashmi et al. [14]	2019	MRCoNS (32.4%)
3.	Jatsho et al. [18]	2020	CoNS (31%), *Klebsiella* (27%), *Acinetobacter* (18.8%)
4.	Afrin et al. [19]	2016	*Staphylococcus aureus* (51.5%), *Escherichia coli* (30.3%)
5.	Pokhrel et al. [20]	2018	*Klebsiella* (33.3%), CoNS (20.3%)
6.	Present Study		CoNS (29.2%), Non-lactose fermenter (19.8%), *Klebsiella* (15.6%)

There are some limitations to this study. Firstly, sample size as well as study time was not optimal to study the less common etiological agents of septicemia. Secondly, there might be an underestimation of the neonates with septicemia because of the low sensitivity of blood culture, especially with the small volume of blood that was collected from neonates. 

## Conclusions

This study provides valuable insights into the clinico-etiological profile and antibiotic sensitivity patterns of bacterial isolates in neonatal septicemia. It is apparent from our study that gram-negative organisms and CoNS were the leading cause of neonatal septicemia in the study population and many were sensitive to very few antibiotics. The distinct microbial profiles associated with EONS and LONS underscore the need for tailored empirical therapy based on the timing of sepsis onset and local epidemiological trends. These findings underscore the importance of vigilant surveillance, rational antimicrobial prescribing practices, and ongoing research efforts to combat the evolving challenges posed by neonatal sepsis. By investigating both EONS and LONS cases, we have shed light on the distribution pattern of causative organisms and their antibiotic susceptibility profiles, offering valuable insights into effective management strategies.
